# Sound trapping in an open resonator

**DOI:** 10.1038/s41467-021-25130-4

**Published:** 2021-08-10

**Authors:** Lujun Huang, Yan Kei Chiang, Sibo Huang, Chen Shen, Fu Deng, Yi Cheng, Bin Jia, Yong Li, David A. Powell, Andrey E. Miroshnichenko

**Affiliations:** 1 School of Engineering and Information Technology, University of New South Wales, Canberra, ACT Australia; 2 Institute of Acoustics, Tongji University, Shanghai, People’s Republic of China; 3 Department of Mechanical Engineering, Rowan University, Glassboro, NJ USA

**Keywords:** Mechanical engineering, Fluidics, Acoustics

## Abstract

The ability of sound energy confinement with high-quality factor resonance is of vital importance for acoustic devices requiring high intensity and hypersensitivity in biological ultrasonics, enhanced collimated sound emission (i.e. sound laser) and high-resolution sensing. However, structures reported so far have been experimentally demonstrated with a limited quality factor of acoustic resonances, up to several tens in an open resonator. The emergence of bound states in the continuum makes it possible to realize high quality factor acoustic modes. Here, we report the theoretical design and experimental demonstration of acoustic bound states in the continuum supported by a single open resonator. We predicted that such an open acoustic resonator could simultaneously support three types of bound states in the continuum, including symmetry protected bound states in the continuum, Friedrich-Wintgen bound states in the continuum induced by mode interference, as well as a new type-mirror symmetry induced bound states in the continuum. We also experimentally demonstrated their existence with quality factor up to one order of magnitude greater than the highest quality factor reported in an open resonator.

## Abstract (web summary)

Though bound states in the continuum (BICs) in acoustic systems are attractive for acoustic resonators design, acoustic BICs typically show low Q-factor. Here, the authors report a high performance open acoustic resonator that supports symmetry-protected, Friedrich-Wintgen and mirror symmetry-induced BICs.

## Introduction

Acoustic resonators constitute the fundamental building block for acoustic metamaterials and metasurfaces^1–4^. They have been widely used in various designs, such as acoustic absorbers and wavefront engineering^5,6^. Typically, most of the reported fabricated acoustic resonators so far exhibited relatively low *Q*-factors, limited to few tens, which may hinder their applications as acoustic sources (e.g., sound laser) and sensors. As a particular type of resonance, bound states in the continuum (BICs)^7,8^, also referred to as embedded trapped modes, have triggered extensive interest within the photonic community because they support zero radiative decay rate and infinite *Q*-factor^9–18^, allowing for enhanced light–matter interaction^19–23^. The history of BIC in acoustics can be traced back to 1951, where Ursell theoretically demonstrated the existence of trapped modes in a sphere that is connected to a cylindrical waveguide on both sides^24,25^. Later, different types of acoustic BICs, including symmetry-protected BICs, Friedrich–Wintgen BICs, and Fabry–Perot BICs, have been subsequently found theoretically in different kinds of acoustic systems^26–34^. On the experimental side, the existence of symmetry-protected BIC has been confirmed in various systems (such as a plate inside the waveguide or asymmetric pair resonators placing at the end of the waveguide)^35–39^. The measured *Q*-factor of such a BIC, however, was only 40–50.

**Fig. 1:**
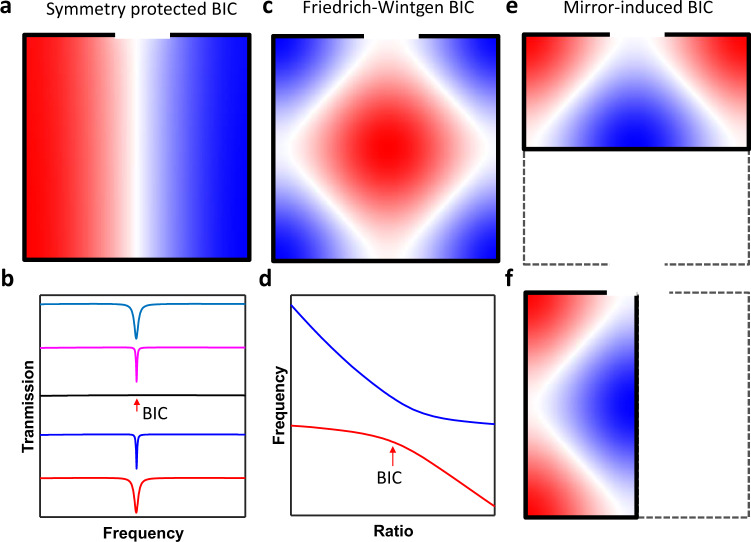
BIC in an open acoustic resonator. **a** Symmetry-protected BIC. **b** Transmission spectra evolution as structure asymmetry is introduced. When the asymmetry ratio decreases to 0 (black curve), ideal BIC occurs. **c** Friedrich–Wintgen BIC induced by mode interferences. **d** Resonant frequency of high-*Q* and low-*Q* modes as a function of size ratio of the open resonator. **e** Mirror-induced BIC for mirror along the *x*-axis. **f** Mirror-induced BIC for mirror along the *y*-axis.

In this work, we demonstrate that an open acoustic resonator could simultaneously support three types of BICs, including symmetry-protected BICs (Fig. 1a, b), mode interference-induced BICs (Fig. 1c, d), as well as newly observed mirror-symmetry-induced BICs (Fig. 1e, f). We also experimentally confirm the existence of these BICs. The measured *Q*-factors for these BICs are up to 250, 583 and 393, respectively. To the best of our knowledge, these are the largest *Q*-factors of acoustic resonances reported so far.

## Results

**Fig. 2:**
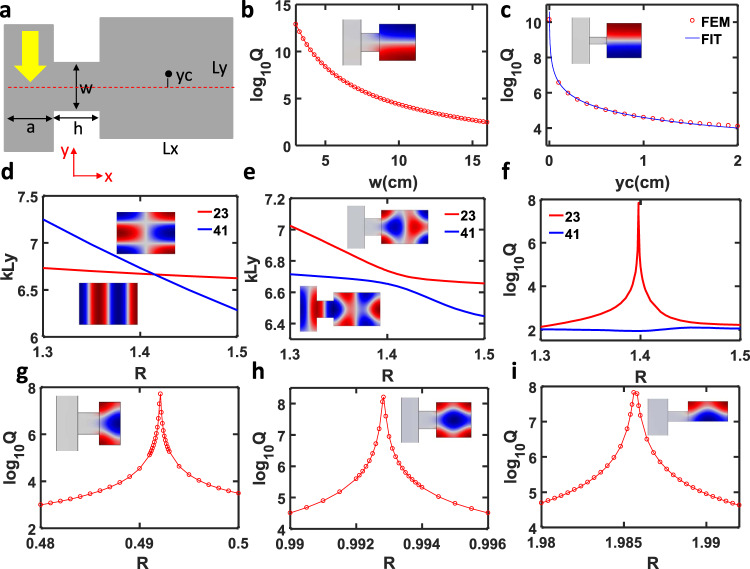
BIC in an open acoustic resonator. **a** Schematic drawing of an open acoustic resonator, where an acoustic waveguide is coupled to a rectangular resonator. **b** *Q*-factor of mode M_12_ vs. neck width. **c** *Q*-factor of mode M_12_ vs. *y*_c_. **d** eigenfrequency *kLy* of modes M_41_ and M_23_ vs. size ratio in a closed resonator. **e** eigenfrequency *kLy* of modes M_41_ and M_23_ vs size ratio in an open resonator. **f** *Q*-factor of modes M_41_ and M_23_ vs. size ratio in an open resonator. **g** *Q*-factor of mode M_13_ in a half-open resonator. **h** *Q*-factor of mode M_13_ in a full resonator. **i** *Q*-factor of mode M_13_ in another half resonator.

We start by investigating the eigenmode properties of the Helmholtz-like resonator system shown in Fig. 2a. Such an open system can be treated as a closed rectangular acoustic cavity subject to deformation. A small air gap is opened to introduce the interaction between the cavity and the exterior environment. The role of the acoustic waveguide connecting to the neck is to guide the incoming acoustics waves, enabling measurement in experiments, as will be described in the later section. Because of its non-Hermitian nature, this open system turns the closed cavity modes into leaky modes (also denoted as quasi-normal modes). The leaky modes have complex eigenfrequencies *ω* = *ω*_0_−*iγ*, where *ω*_0_ and *γ* are the resonant frequency and radiative decay rate, respectively. The radiative *Q*-factor can be derived from *Q* = *ω*_0_/(2*γ*). Here, each leaky mode’s complex eigenfrequency is calculated by COMSOL Multiphysics. Following the definition in ref. ^29^, the leaky modes are labelled as *M*_*pq*_, where *p* and *q* are the number of maxima in the pressure field along the *x*- and *y*-axes, respectively.

We demonstrate that the reflection or transmission spectrum of such Helmholtz resonators can be perfectly reproduced with a complex eigenfrequency of leaky modes based on coupled-mode theory (CMT)^40^ (see Supplementary Note 1 and Supplementary Fig. 1 in supporting material (SM)). Thus, the goal in searching BICs is to find leaky modes with infinitely large *Q*-factor. Since a BIC is also accompanied by vanishing linewidth of the acoustic resonance in the reflection or transmission spectrum^41^, we calculate the reflection coefficient as a function of frequency and size ratios in Supplementary Fig. 2 while assuming the system is lossless. Many BICs can be found by checking the vanishing linewidth of resonant peaks. These BICs can be categorized into three types (see Fig. 1): symmetry protected BICs (Fig. 1a, b), Friedrich–Wintgen BICs induced by two-mode interference (Fig. 1c, d) and mirror-effect induced BICs (Fig. 1e, f). Except where explicitly mentioned, only the lossless case is studied for calculating the leaky modes. The realistic system’s losses will deteriorate the total quality factor by 1/*Q* = 1/*Q*_abs_ + 1/*Q*_rad_ and will be discussed in the experiment section.

### Symmetry-protected quasi-BIC

Referring to Fig. 2a, we consider a rectangular cavity of dimensions *Lx* by *Ly*. If only one open port is introduced on the left of the acoustic resonator, symmetry is broken along the *y*-axis but still maintained along the *x*-axis. Thus, it will support several types of symmetry protected BICs as long as the centre of the neck and resonator are symmetric with respect to the *x*-axis. The left waveguide’s existence will further lead to broken symmetry along the *x*-axis, and thus turn an ideal BIC into a quasi-BIC. Without loss of generality, we use mode M_12_ in the square cavity as an example to describe the effect of neck width *w* and centre shift *y*_c_ on the *Q*-factor of BIC. Interestingly, from Fig. 2b, the *Q* factor is still larger than 10^4^ for the system with protected symmetry along the *y*-axis even when the neck’s width is half of the right cavity width. The *Q*-factor increases exponentially with decreasing neck width. This can be understood by treating the neck as a perturbation of the square cavity. The smaller the neck width, the smaller the perturbation, and thus the larger the *Q*-factor is. Another interesting finding is that the *Q*-factor is proportional to 1/(*y*_c_)^2^ when the neck width is small compared to the width of the right cavity, as shown in Fig. 2c. This phenomenon has been observed and proved for BIC in photonic systems^42^. Additional symmetry protected quasi-BICs can be found in such structures as long as *q* is an even number for mode *M*_*pq*_ (see Supplementary Fig. 3).

### Friedrich–Wintgen BIC induced by mode interference

Friedrich and Wintgen demonstrated that in quantum mechanics full destructive interferences of two degenerate modes give rise to the avoided crossing of eigenvalues, accompanied by the formation of a BIC^43,44^. This type of BIC can be easily constructed in our system by tuning the size ratio of a rectangular cavity. For a closed rectangular cavity, cavity modes *M*_*pq*_ and *M*_*p*+2,*q*−2_ will become degenerate at a certain size ratio. When an air gap is introduced on one side of the rectangular cavity, strong coupling between these two modes results in the giant enhancement of *Q*-factor for one mode but suppresses it for another. We use paired modes M_23_ and M_41_ as an example to illustrate this principle. The pressure distributions for these two modes are shown in the inset of Fig. 2d. It is found that the eigenfrequencies for these two modes cross at *R* = *Lx*/*Ly* = 1.42 in a closed cavity. All the eigenmodes become leaky modes with complex eigenfrequencies when introducing the neck to couple the acoustic waveguides to the cavity. Based on the eigenmodes analysis, we find that the real part of eigenfrequencies exhibits an avoided crossing. Simultaneously, the *Q*-factor for mode M_23_ is enhanced to a maximum of 6.87 × 10^7^ but suppressed to a minimum of 86.76 for mode M_41_ at *R* = 1.398, as shown in Fig. 2e, f.

Moreover, the avoided crossing suggests that these two modes interchange with each other after the size ratio passes through the critical size ratio. This exciting phenomenon is confirmed by the mode evolution, as shown in Supplementary Fig. 4. Indeed, the upper branch mode, for example, evolves from mode M_23_ into mode M_41_ when the size ratio increases from 1.3 to 1.5. Following a similar strategy, more BICs induced by mode interference, such as M_13_−M_31_ and M_33_−M_51_, can be found by merely constructing avoided crossing (see Supplementary Fig. 5a–d). Besides, we find that BIC can also be found in mode crossing for M_24_ and M_42_ (see Supplementary Fig. 5e, f). Here, we emphasize that not all crossings of two modes of a closed cavity can bring about BICs. The two modes must have the same parity along both *x*- and *y*-directions. This is also the reason why we choose paired modes *M*_*pq*_ and *M*_*p*+2,*q*−2_ here.

### Mirror-induced BIC

In addition to the abovementioned two types of BICs that have been intensively studied in the photonics community, we also find a new type of BIC: mirror-symmetry-induced BIC (see Fig. 1e, f). Because all the outer boundary conditions are set as a hard wall in simulation, we can view the rightmost boundary as a partial mirror. All the eigenmodes with almost symmetric pressure distribution in the full-size resonator can also be found in a half-size resonator. Thus, many other BICs can also be constructed by simply shrinking the width to half based on this mirror effect. For example, one can easily find a BIC at *R* = 0.498 for mode M_13_ (Fig. 2g), which is indeed half of the critical size ratio for BIC M_13_ in a full resonator (Fig. 2f).

Moreover, we find that the mirror effect also occurs for the *x*-axis. As shown in Fig. 2i, a BIC can be found at *R* = 1.986. Following a similar approach, more BICs can be constructed (see Supplementary Figs. 6 and 7). The mirror effect indicates that one can achieve extreme pressure confinement even with reduced size in the 2D case, suggesting an effective way to engineer the Purcell factor that is the key to realize enhanced acoustic emission^45^.

Note that the above three BIC types are not limited to regular rectangular shaped resonators. We can also find them in the elliptical resonator (see Supplementary Fig. 8). The only difference is that the size ratio is defined as *R* = *a*/*b*, where *a* and *b* are semi-major and semi-minor axes, respectively. Besides, the conclusion drawn in the 2D case can be straightforwardly generalized to three-dimensional (3D) open resonators (e.g. cuboid resonators) (see Supplementary Figs. 9–11). More freedom is provided in the 3D case because three parameters including length, width and height, are involved in the mode calculation.

### Experimental verification of BIC

**Fig. 3:**
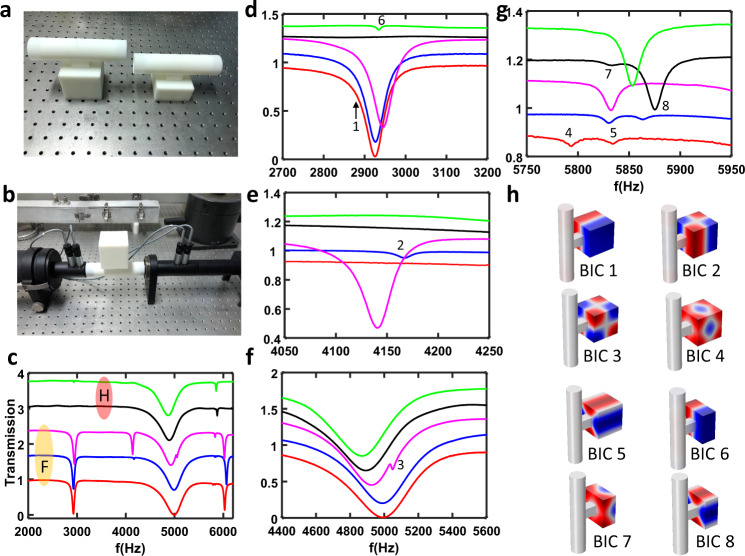
Experimental verification in an open resonator. **a** Image of 3D printed full (left) and half (right) acoustic resonator. **b** Transmission measurement system. **c** Full transmission spectrum in the frequency range 2000–6000 Hz. Red, blue and magenta lines represent full resonator (*Lx* = *Ly* = *Lz* = 60 mm) with *y*_c_ = *z*_c_ = 0 mm, *y*_c_ = 1 mm and *z*_c_ = 0 mm, and *y*_c_ = *z*_c_ = 4 mm, respectively; Black and green lines represent half resonator (*Lx* = 30 mm, *Ly* = *Lz* = 60 mm) with y_c_ = *z*_c_ = 0 mm, and *y*_c_ = 1 mm and *z*_c_ = 0 mm. Eight BICs are observed and labelled as 1–8. **d–g** zoom in transmission spectrum in the frequency range 2700–3200 Hz (**d**), 4050–4250 Hz (**e**), 4400–5600 Hz (**f**), and 5750–5950 Hz (**g**). **h** pressure field distribution of eight BICs in the full and half resonator, which is also correlated to peaks 1–8 in the transmission spectrum.

**Fig. 4:**
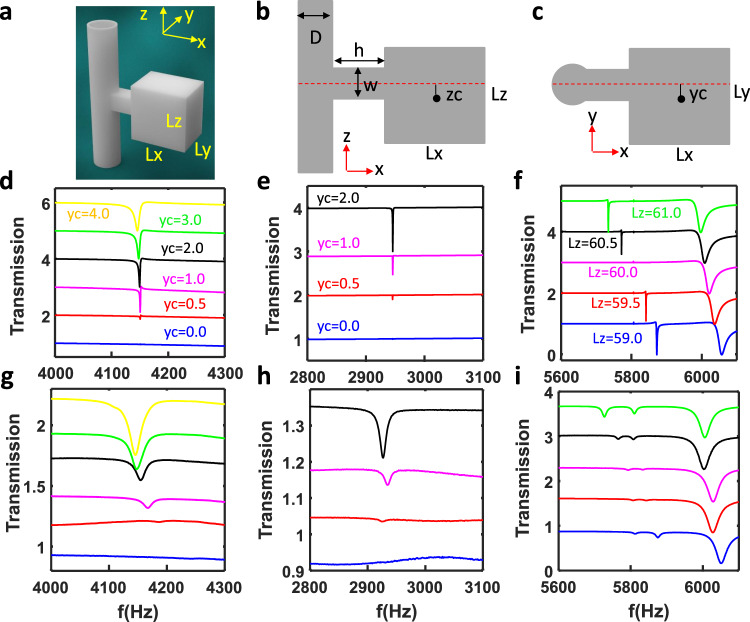
Transmission spectra of an open resonator. **a** Schematic drawing of the 3D open resonator. **b** Cross-section in XoZ plane for the 3D open resonator. **c** Cross-section in *X*o*Y* plane for the 3D open resonator. **d**–**f** Simulated transmission spectra for full resonator (*Lx* = *Ly* = *Lz* = 60 mm) with different *y*_c_ (**d**), half resonator (*Lx* = 30 mm, *Ly* = Lz = 60 mm) with different *y*_c_ (**e**) and full resonator (*Lx* = *Ly* = 60 mm) for different values of *Lz* (**f**). **g**–**i** Measured transmission spectra for full resonator (*Lx* = *Ly* = *Lz* = 60 mm) with different *y*_c_ (**g**), half resonator (*Lx* = 30 mm, *Ly* = *Lz* = 60 mm) with different *y*_c_ (**h**) and full resonator (*Lx* = *Ly* = 60 mm) for different values of *Lz* (**i**).

Next, we switch to the experimental demonstration of all three types of BICs. We fabricate two acoustic cuboid resonators shown in Fig. 3a: full resonator and half resonator. In experiments, the left circular tube’s diameter is fixed as *d* = 29 mm while the length, width, and height for the neck are set as 40, 20, 20 mm, respectively. Figure 3b shows the measurement set up while Fig. 4a–c depicts the structure’s schematic. Figure 3c shows the measured transmission spectra for the full resonator and half resonator, and Fig. 3d–g corresponds to the zoomed-in range in the vicinity of the BICs while Fig. 3h shows the pressure distribution of BICs. The excellent agreement can be found between simulation and experiment over the full spectrum (see Supplementary Fig. 12). Other leaky modes that are not BICs are given in Supplementary Fig. 13. In the following, we discuss all three types of BIC observed in experiments.

To study the symmetry protected BIC, we fix *Lx* = *Ly* = *Lz* = 60 mm (*Lx* = 30 mm, *Ly* = *Lz* = 60 mm) for full (half) resonator and only change the centre shift *y*_c_ and *z*_c_ for the cuboid resonator with respect to the symmetric axis of the neck. From Fig. 3d–f, it can be found that there is three symmetry protected BICs. For example, when *y*_c_ increases from 0 mm to 1 mm for the half resonator, a dip shows at around 2920 Hz (green curve), which corresponds to mode M_121_ (see Fig. 3h-BIC 6). However, we did not observe M_121_ (BIC 1) for the full resonator when the same asymmetry parameter *y*_c_ = 1 mm is introduced. This can be explained by the fact that this mode’s *Q*-factor decreases relatively slowly with respect to *y*_c_ and makes the resonance vanish in the low-*Q* mode background spectrum. Therefore, further enlarging *y*_c_ may help to excite this mode.

**Fig. 5:**
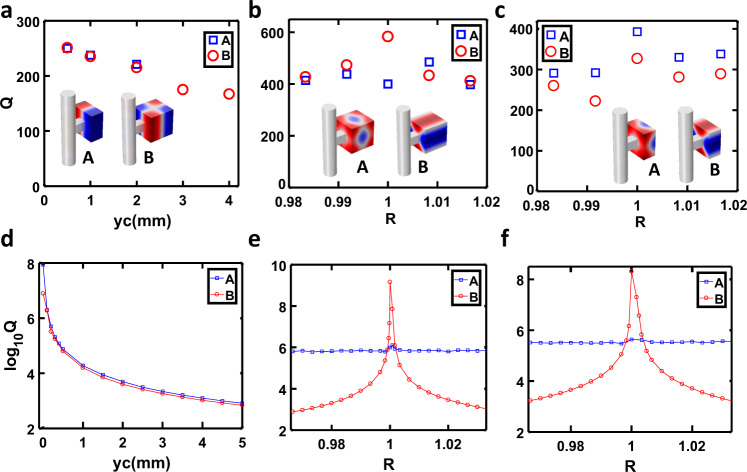
*Q*-factor of QBIC. **a** Measured *Q*-factor of modes A (M_121_) and B (M_221_) for the half resonator and full resonator as a function of *y*_c_. **b** Measured *Q*-factor of modes A (M_131_) and B (M_113_) for full resonator as a function of size ratio. **c** Measured *Q*-factor of modes A (M_131_) and B (M_113_) for half resonator as a function of size ratio. **d** Calculated *Q*-factor of modes A (M_121_) and B(M_221_) for the half resonator and full resonator as a function of *y*_c_. **e** Calculated *Q*-factor of modes A (M_131_) and B (M_113_) for full resonator as a size ratio function. **f** Calculated *Q*-factor of modes A (M_131_) and B (M_113_) for half resonator as a size ratio function.

Interestingly, another symmetry protected BIC M_221_ (BIC 2) appears for a full resonator with *y*_c_ = 1 mm (blue curve), evidenced by a shallow dip in the transmission spectrum (see Fig. 3e). For mode M_222_ (BIC 3), it is not enough to introduce the asymmetry by shifting *y*_c_. The mode becomes visible in the transmission spectrum when *y*_c_ and *z*_c_ are adjusted to 4 mm simultaneously, as shown in Fig. 3f (magenta curve). We also systematically study the role of *y*_c_ (or *y*_c_ = *z*_c_) on the *Q* factor for BIC mode M_121_, M_221_ and M_222_. The simulated and measured transmission spectra for the modes M_121_ and M_221_ are presented in Fig. 4d, e and g, h, respectively while the cases of M_222_ can be found in Supplementary Fig. 14. Good agreement can be found between these two. When *y*_c_ is reduced to zero, the vanishing line width of resonances indicates the BIC’s appearance. The *Q*-factor can be obtained by fitting the spectrum with the Fano formula^46^ (see Supplementary Note 2 and Supplementary Fig. 15). Figure 5a, b shows the measured *Q*-factor vs. *y*_c_ for the former two modes while the *Q*-factor of M_222_ is put in Supplementary Fig. 16. The maximum *Q*-factor for these two modes is only 250, lower than the theoretical prediction (see Fig. 5e, f). This is because there are losses in the real system due to the thermo-viscous boundary layers, which degrades the *Q*-factor. Moreover, the *Q*-factor reduces with the increasing *y*_c_, matching the trend of *Q*-factor vs. *y*_c_. Here, it is worth noting that the *Q*-factor for *y*_c_ = 0 mm should be larger than 250. However, we cannot retrieve the exact value because they are almost indistinguishable from the background, which is exactly the signature of BIC.

For the two-mode interference induced BIC, we fix *Lx* = *Ly* = 60 mm and *y*_c_ = *z*_c_ = 0 mm, but only vary *Lz*. The size ratio *Rx* and *Ry* are defined as *Rx* = *Lz*/*Lx* and *Ry* = *Lz*/*Ly*, respectively. Thus, we have *R* = *Rx* = *Ry* for *Lx* = *Ly*. For the fixed *Lz* = 60 mm (*Rx* = *Ry* = 1), we can find from Fig. 3g that there are two BICs (BICs 4–5) in the range 5750–5950 Hz, where the pressure field distributions are shown in Fig. 3h (modes 4 and 5 for the full resonator, and modes 7 and 8 for the half resonator). Unlike symmetry-protected BICs, these two BICs always exist regardless of the value of *y*_c_. Their *Q*-factors only depend on the size ratio. The simulated and measured transmission spectra for full resonator are presented in Fig. 4f and i where resonator height *Lz* varies from 59.5 mm to 60.5 mm. The measurement results of the half resonator with different *Lz* can be found in Supplementary Fig. 17. Figure 4b, c shows the *Q*-factor of two modes as functions of size ratio. For the full resonator case, the maximum *Q*-factor is 583 at *R* = 1 for mode B(M_113_) while it is 485 at *R* = 1.008 for mode A (M_131_). Note that the trend of measured *Q* factor vs. *R* devitates from the theoretical prediction (see Fig. 5e, f) because of inevitable intrinsic loss in the real system. The theoretical calculated *Q*-factor for mode A is maintained at a high value because the size ratio *R*_*xy*_ = *Ly*/*Lx* is always 1, which is precisely the critical size ratio. For mode B, the *Q*-factor reaches the maximum at critical size ratio *Rx* = *Ry* = 1. However, the majority of *Q*-factors are ranged between 400–500. Such high-*Q* factors are already good enough for real applications, such as ultra-narrowband acoustic absorbers and enhanced acoustic emission. A similar phenomenon can also be observed for the half resonator. The only difference is that the maximum *Q*-factor for modes A (BIC 4) and B (BIC 5) are 393 and 327, respectively, which are lower than the full resonator case. The reduction of *Q*-factor may be attributed to increased thermo-viscous losses arising from the acoustic resonator’s smaller volume.

## Discussion

We report the theoretical design of three types of acoustic BICs, including symmetry-protected BICs, modes interference-induced BICs and mirror-symmetry-induced BICs in a simple open resonator. Different types of BICs can be induced by tuning the resonators’ geometrical parameters. Following the design principle, we fabricate such acoustic resonators and experimentally demonstrate these BICs by measuring the transmission spectrum. We found that the largest *Q*-factors retrieved from the transmission spectrum are 250, 583 and 393 for symmetry-protected QBIC, modes interference induced QBIC and mirror-symmetry induced QBIC. In comparison to the previous work, the measured *Q*-factor in this work shows one order of magnitude enhancement. Since the high-*Q* acoustic resonance is always accompanied by the large pressure enhancement, it is expected that our design may find promising applications in enhanced acoustic emissions and may lead to the practical realization of the acoustic laser. Also, the high-*Q* nature of QBIC makes it possible to build an acoustic filter and sensor with superior performance. Hence, we envision that our design may bring more opportunities in enhancing acoustic-matter interactions.

## Methods

### Numerical simulations

All simulations in this paper are performed with the commercial software COMSOL Multiphysics. The speed of sound and air density is 349 m/s (corresponding to the experimental temperature of 30 °C) and 1.29 kg/m^3^, respectively. When calculating the eigenmodes and transmission (or reflection spectrum), we apply perfect matched layer boundaries at the two ends of waveguides to mimic acoustic wave propagation in the infinite space. The other exterior boundaries are set as rigid.

### Device fabrication and measurement

The experimental samples are fabricated by 3D-printing technology using laser sintering stereolithography (SLA, 140 μm) with a photosensitive resin (UV curable resin), exhibiting a manufacturing precision of 0.1 mm. The complex transmission (and reflection) coefficients of the samples are measured using a Brüel & Kjær type-4206T impedance tube with a diameter of 29 mm. A loudspeaker generates a plane wave, and the amplitude and phase of local pressure are measured by four 1/4-inch condenser microphones (Brüel & Kjær type-4187) situated at designated positions. The complex transmission (and reflection) coefficients are obtained by the transfer matrix method.

## Supplementary information

Supplementary Information

## Data Availability

All data are available in the main text or the Supplementary materials.

## Code availability

The code used for the analyses will be made available upon e-mail request to the corresponding author.
