# Supplementary material for: Sound trapping in an open resonator
Source: Nat Commun. 2021 Aug 10;12:4819. doi: 10.1038/s41467-021-25130-4 (PMC8355331; doi:10.1038/s41467-021-25130-4)
Supplement: Supplementary file 1 — Supplementary Information [file 41467_2021_25130_MOESM1_ESM.docx]

**Supporting Information for Sound Trapping in an Open Resonator**

**Lujun Huang^1#^ , Yan Kei Chiang^1#^, Sibo Huang^2#^, Chen Shen^3#^, Fu Deng^1^, Yi Cheng^2^, Bin Jia^2^, Yong Li^2*^, David A Powell^1*^ and Andrey E Miroshnichenko^1*^**

*^1^ School of Engineering and Information Technology, University of New South Wales, Canberra,*

*Northcott Drive, ACT, 2600, Australia*

*^2^Institute of Acoustics,Tongji University, Shanghai, 200092, People’s Republic of China*

*^3^Department of Mechanical Engineering, Rowan University, Glassboro, NJ, 08028, USA*

# These authors contributed equally to this work

*^*^ yongli@tongji.edu.cn,* [*david.powell@adfa.edu.au*](mailto:david.powell@adfa.edu.au)*, andrey.miroshnichenko@unsw.edu.au*

**Supplementary Note 1-Coupled Mode Theory for Acoustic Resonator with Two Ports**

Here, we consider single mode acoustic resonator with two ports^1^, as shown in Fig.S1a. The dynamic equation for amplitude a of resonance is given as

$\frac{da}{dt}=(-i{}_{0}-)a+\left( \begin{matrix} k_{1} & k_{2} \end{matrix} \right)\left( \begin{matrix} S_{1+} \\ S_{2+} \end{matrix} \right)$ (1)

$\left( \begin{matrix} S_{1-} \\ S_{2-} \end{matrix} \right)=C\left( \begin{matrix} S_{1+} \\ S_{2+} \end{matrix} \right)+a\left( \begin{matrix} d_{1} \\ d_{2} \end{matrix} \right)$ (2)

Where ω_0_ and γ are resonant frequency and radiative decay rate of acoustic mode, respectively. The amplitude a is normalized such that |a|^2^ represents the energy inside the resonator. Since there are two ports for such a system, the resonant mode is excited by the incoming wave S_1+_ and S_2+_, and then coupled out as outgoing wave S_1-_ and S_2-_. Except for resonant assisted coupling between two ports, the incoming wave and outgoing wave is also connected by the direct scattering matrix C.

The scattering matrix S can be obtained as

$\left( \begin{matrix} S_{1-} \\ S_{2-} \end{matrix} \right)=S\left( \begin{matrix} S_{1+} \\ S_{2+} \end{matrix} \right)=[C+\frac{1}{i\left( {}_{0}- \right)+}\left( \begin{matrix} d_{1}k_{1} & d_{1}k_{2} \\ d_{2}k_{1} & d_{2}k_{2} \end{matrix} \right)]\left( \begin{matrix} S_{1+} \\ S_{2+} \end{matrix} \right)$ (3)

According to energy conservation, we will have

${d_{1}d}_{1}^{*}{{+d}_{2}d}_{2}^{*}=$ (4)

Applying time reversal symmetry gives us

$k_{1}=d_{1}, k_{2}=d_{2}$ (5)

$C\left( \begin{matrix} d_{1}^{*} \\ d_{2}^{*} \end{matrix} \right)=-\left( \begin{matrix} d_{1} \\ d_{2} \end{matrix} \right)$ (6)

The scattering matrix S can be proved as a unitary matrix.

If we choose the reference plane that make structure on both sides symmetrical with respective to mirror plane, two diagonal elements of the scattering matrix must be equal. Then we will have

$d_{1}^{2}=d_{2}^{2}$ (7)

Matrix C is also unitary and should take the following form

$C=e^{i}\left( \begin{matrix} \cos\theta& i\sin\theta\\ i\sin\theta& \cos\theta\end{matrix} \right)$ (8)

Where φ and θ are real constants.

Based on eq. (4) and eq. (6), we can obtain *d_1_* and *d_2_*. The scattering matrix S for the whole system can be expressed as

$S=e^{i}[C+\frac{}{i\left( {}_{0}- \right)+}\left( \begin{matrix} -(\cos\theta\pm i\sin\theta) & \mp(\cos\theta\pm i\sin\theta) \\ \mp(\cos\theta\pm i\sin\theta) & -(\cos\theta\pm i\sin\theta) \end{matrix} \right)]$ (9)

Where the sign ± represents even (odd) mode with respective to the mirror plane (*d_1_*=+(-)*d_2_*).

From eq. (9), we can obtain the reflection coefficient R as

$R=\frac{{(\omega-\omega_{0})}^{2}\cos^{2} \theta+{}^{2}\sin^{2} \theta\mp2\sin\theta\cos\theta(\omega-\omega_{0})}{{(\omega-\omega_{0})}^{2}+{}^{2}}$ (10)

Thus, the reflection (transmission) coefficient R (T=1-R) can be directly predicted with complex eigenfrequency of leaky modes. Note that when θ=0, π/2, π, 3π/2, R(T) has a symmetric Lorentz line-shape. Otherwise, Fano resonance line-shape occurs in R(T).

**Supplementary Note 2-Fano resonance fitting of measured transmission spectrum**

Since transmission spectrum shows Fano resonance features, we use the following equation to fit the transmission spectrum to extract the resonant frequency and radiative decay rate^2^

$T=T_{\mathrm{bg}}+A\frac{{(q{}_{0}+-{}_{0})}^{2}}{{}_{0}^{2}+{(-{}_{0})}^{2}}$ (11)

in which γ_0_ and ω_0_ are the damping rate and resonant frequency of the mode and q is the Fano parameters, A is the amplitude of the resonance. Then, the Q-factor can be obtained by Q=ω_0_/(2γ_0_).

**Reference**

1. Fan, S., Suh, W. & Joannopoulos, J. D. Temporal coupled-mode theory for the Fano resonance in optical resonators. *J. Opt. Soc. Am. A* **20**, 569–572 (2003).

2. Miroshnichenko, A. E., Flach, S. & Kivshar, Y. S. Fano resonances in nanoscale structures. *Rev. Mod. Phys.* **82**, 2257–2298 (2010).


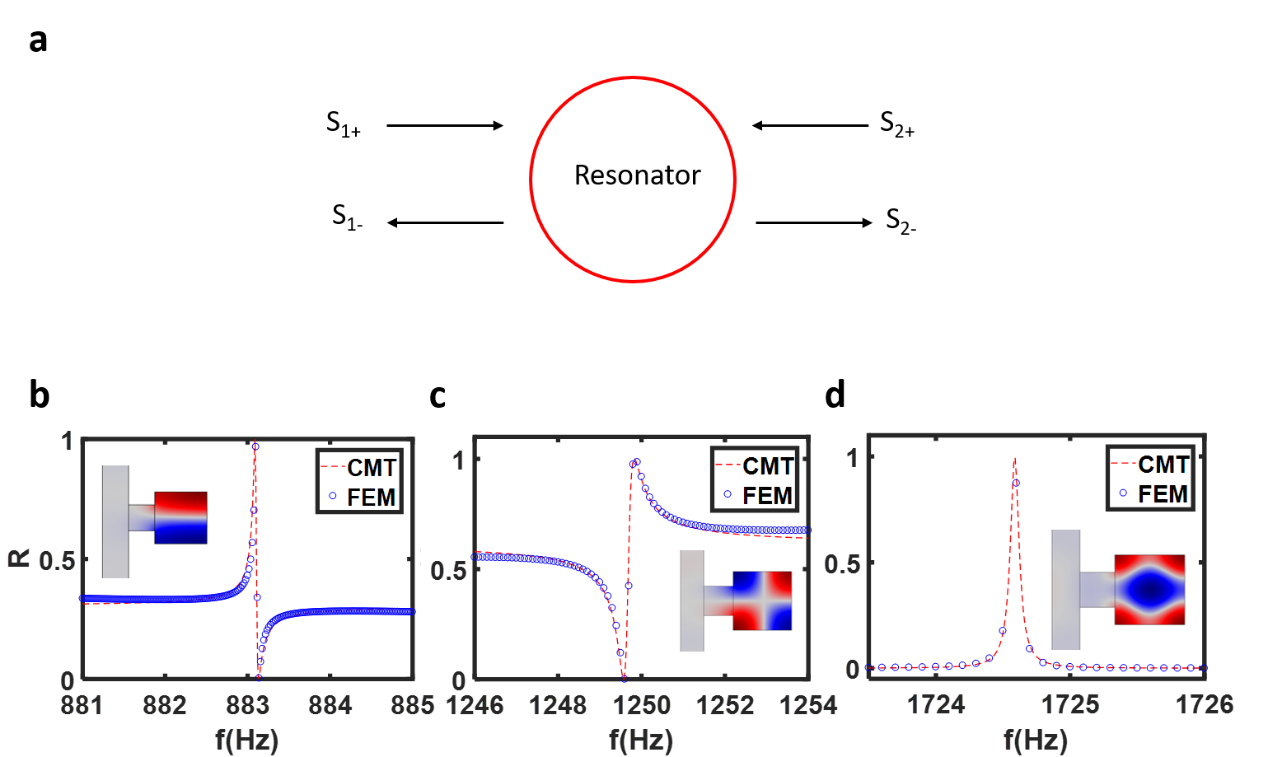


**Supplementary Figure 1. Coupled mode theory.** a, schematics of an acoustic resonator coupled to two ports b-d, reflection spectrum obtained by coupled mode theory and finite element method.


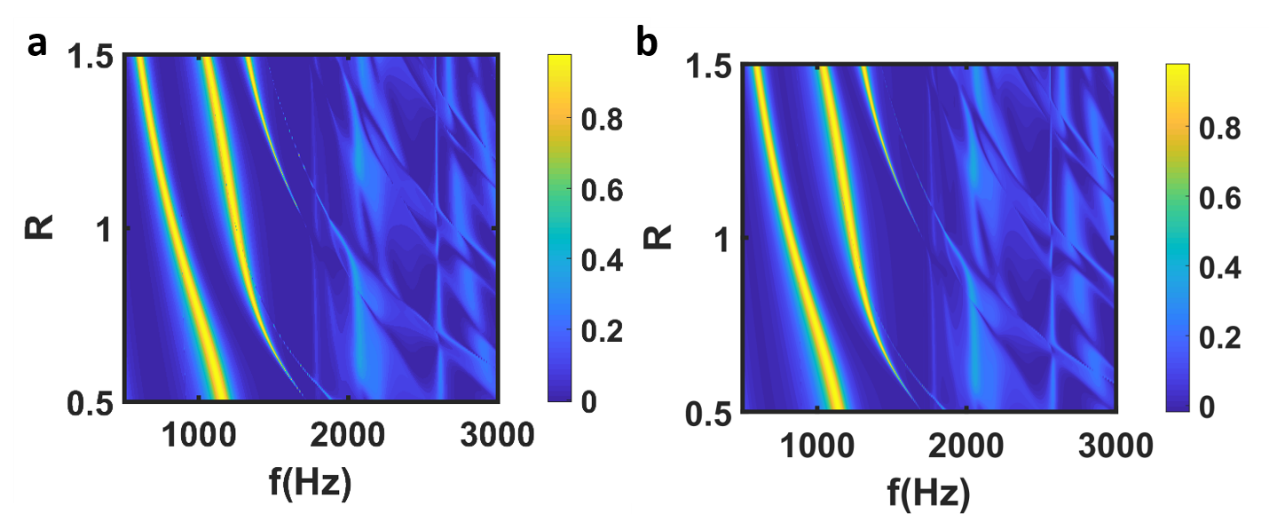


**Supplementary Figure 2. Reflection spectrum mapping vs size ratio.** a, reflection mapping for lossless acoustic resonator. b, reflection mapping for acoustic resonator with thermal viscosity loss.


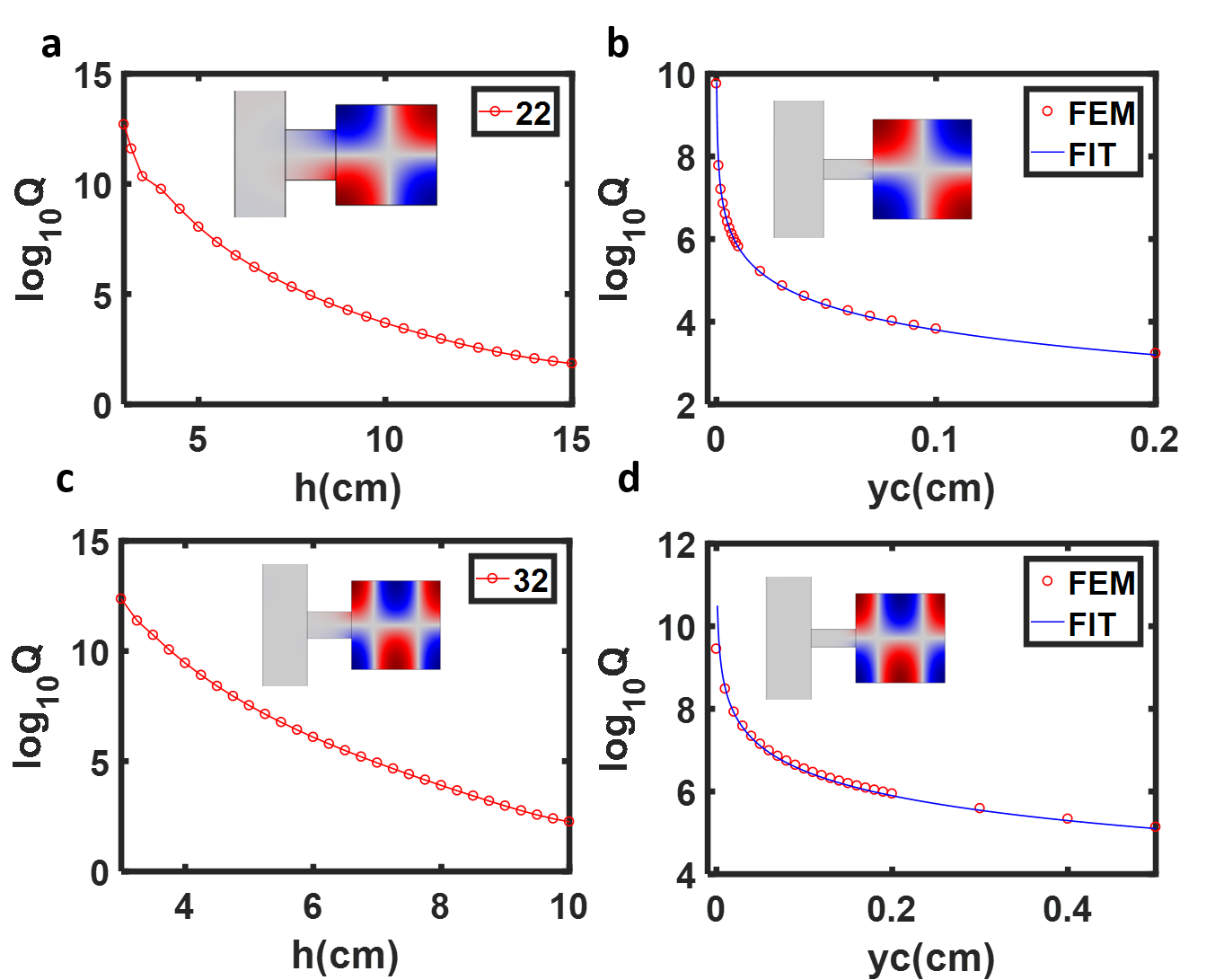


**Supplementary Figure 3. Symmetric protected BIC.** a, Q-factor of mode M_22_ vs neck height. b, Q-factor of mode M12 vs yc. c, Q-factor of mode M_32_ vs neck height. d, Q-factor of mode M_32_ vs yc.


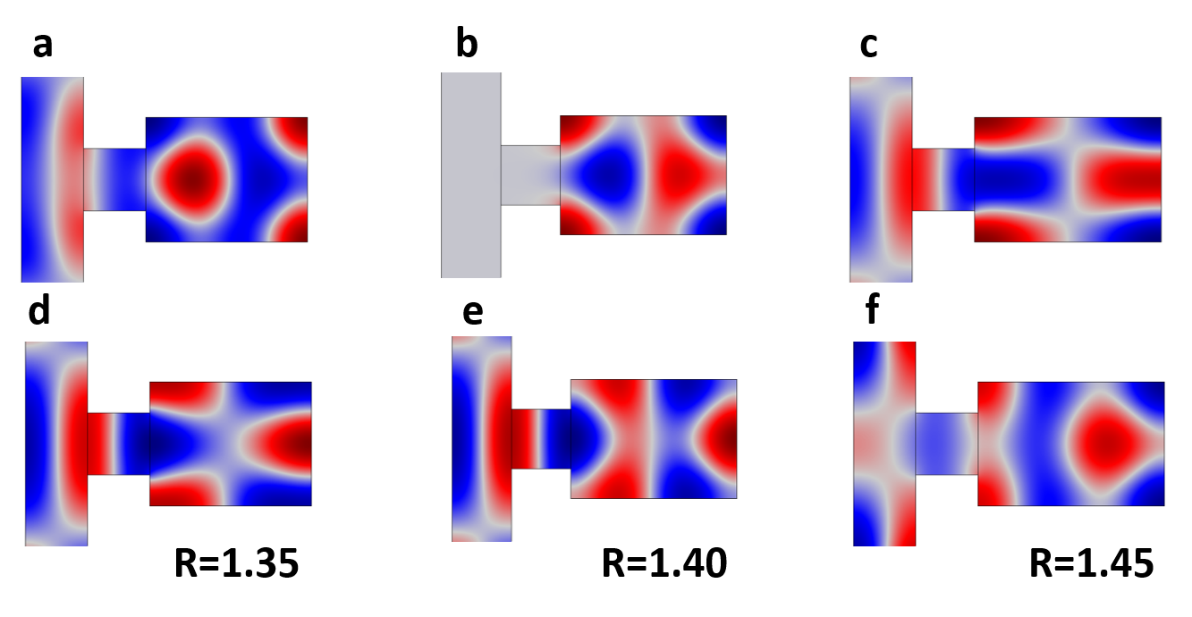


**Supplementary Figure 4. Mode evolution**. a, pressure distribution of M_23_ at R=1.35. b, pressure distribution of M_23_ at R=1.40. c, pressure distribution of M_23_ at R=1.45. d, pressure distribution of M_41_ at R=1.35. e, pressure distribution of M_41_ at R=1.40. f, pressure distribution of M_41_ at R=1.45.


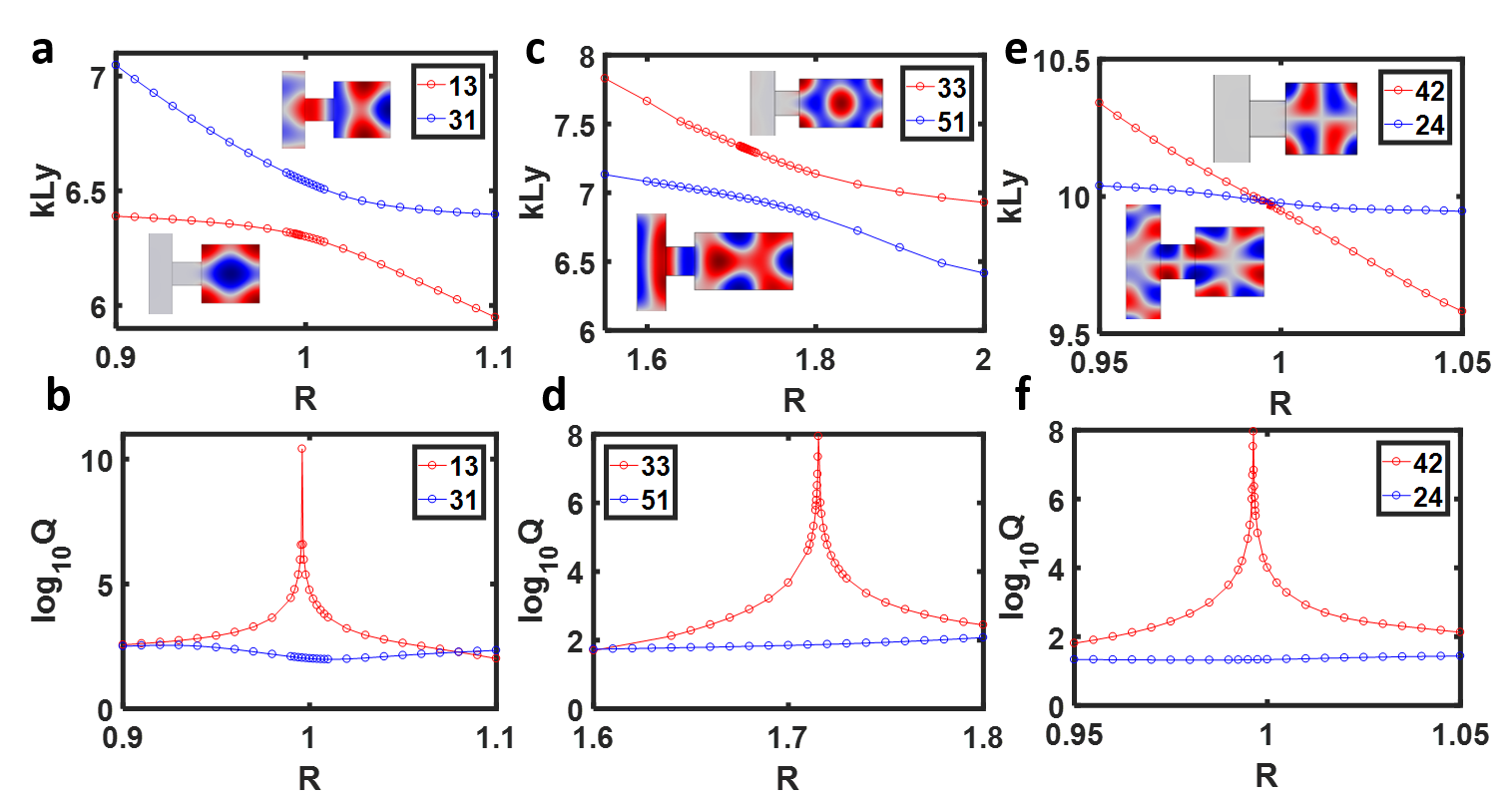


**Supplementary Figure 5. Mode interference induced BIC.** a, eigenfrequency kLy of mode M_13_ and M_31_ vs size ratio in an open resonator. b, Q-factor of mode M_13_ and M_31_ vs size ratio in an open resonator. c, eigenfrequency kLy of mode M_33_ and M_51_ vs size ratio in an open resonator. d, Q-factor of mode M_33_ and M_51_ vs size ratio in an open resonator. e, eigenfrequency kLy of mode M_24_ and M_42_ vs size ratio in an open resonator. f, Q-factor of mode M_24_ and M_42_ vs size ratio in an open resonator.


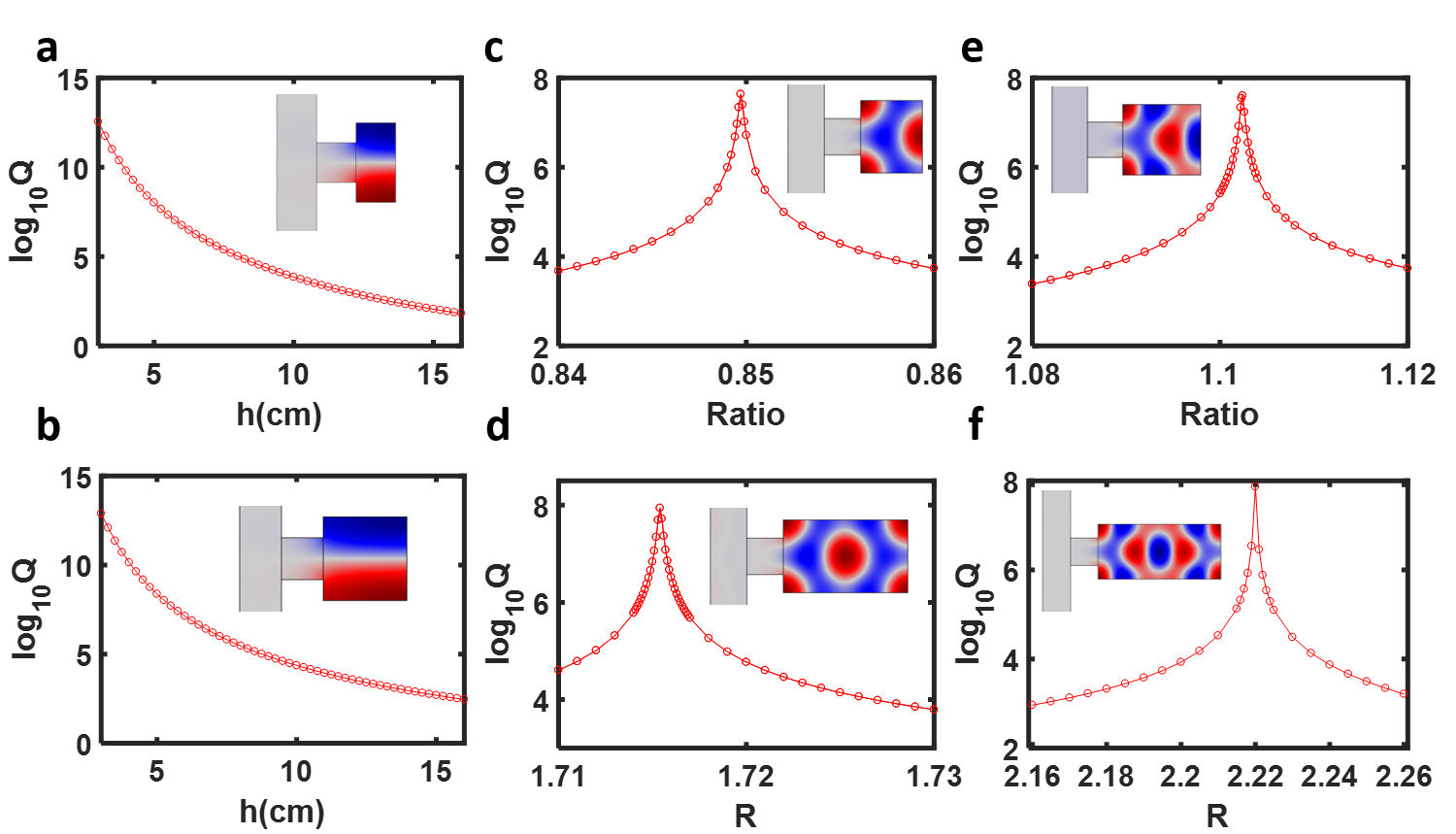


**Supplementary Figure 6. Mirror effect induced BIC in half resonator.** a, Q-factor of mode M_12_ vs neck height in half resonator. b, Q-factor of mode M_12_ vs neck height in full resonator. c, Q-factor of mode M_33_ vs size ratio in half resonator. d, Q-factor of mode M_33_ vs size ratio in full resonator. e, Q-factor of mode M_53_ vs size ratio in half resonator. f, Q-factor of mode M_53_ vs size ratio in full resonator.


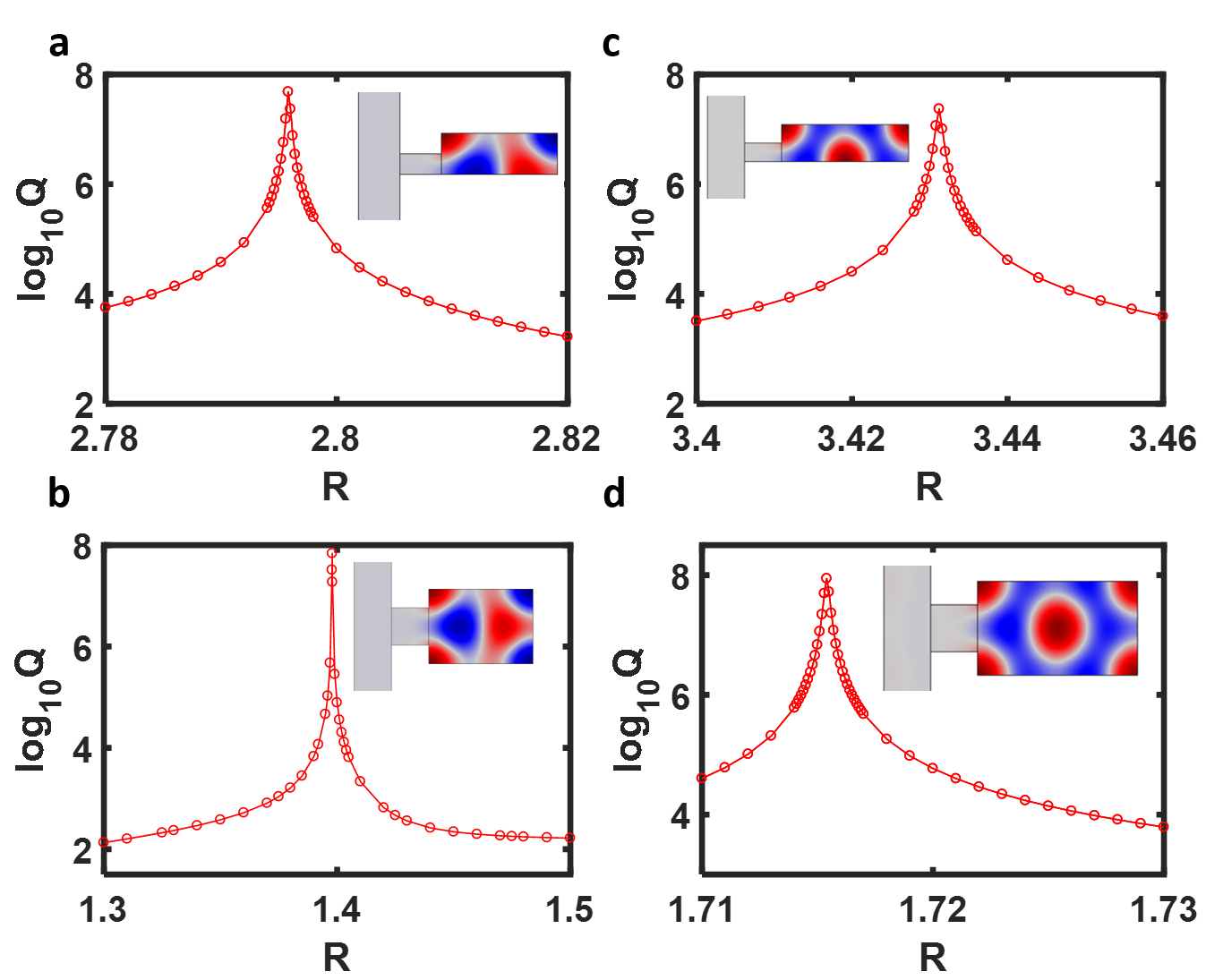


**Supplementary Figure 7. Mirror effect induced BIC in half resonator.** a, Q-factor of mode M_23_ vs size ratio in half resonator. b, Q-factor of mode M_23_ vs size ratio in full resonator. c, Q-factor of mode M_33_ vs size ratio in half resonator. d, Q-factor of mode M_33_ vs size ratio in full resonator.


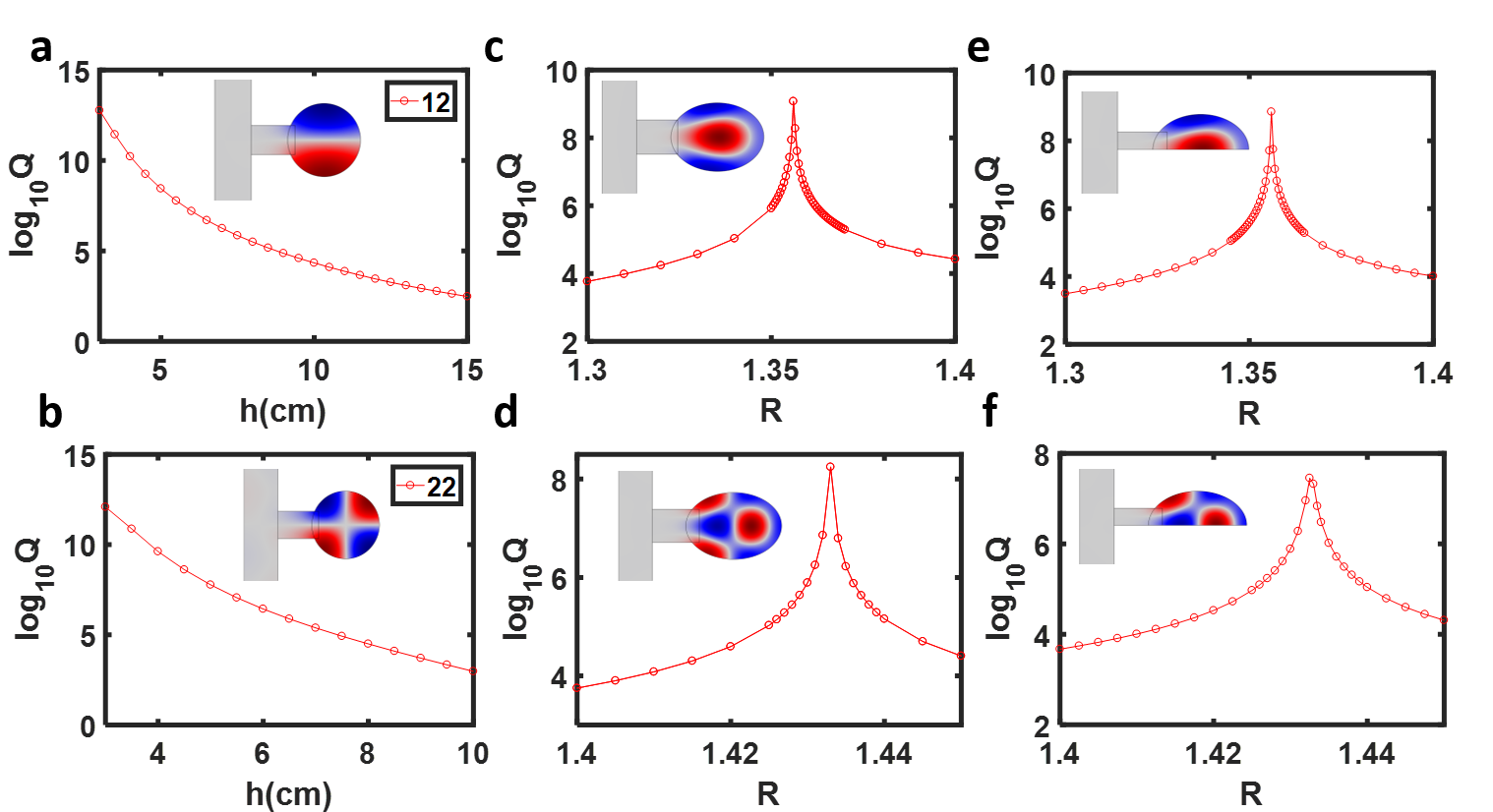


**Supplementary Figure 8. BIC in elliptical resonator.** a, Q-factor of mode M_12_ vs neck height in full resonator. b, Q-factor of mode M_22_ vs neck height in full resonator. c, Q-factor of mode M_13_ vs size ratio in full resonator. d, Q-factor of mode M_23_ vs size ratio in full resonator. e, Q-factor of mode M_13_ vs size ratio in half resonator. f, Q-factor of mode M_23_ vs size ratio in half resonator.


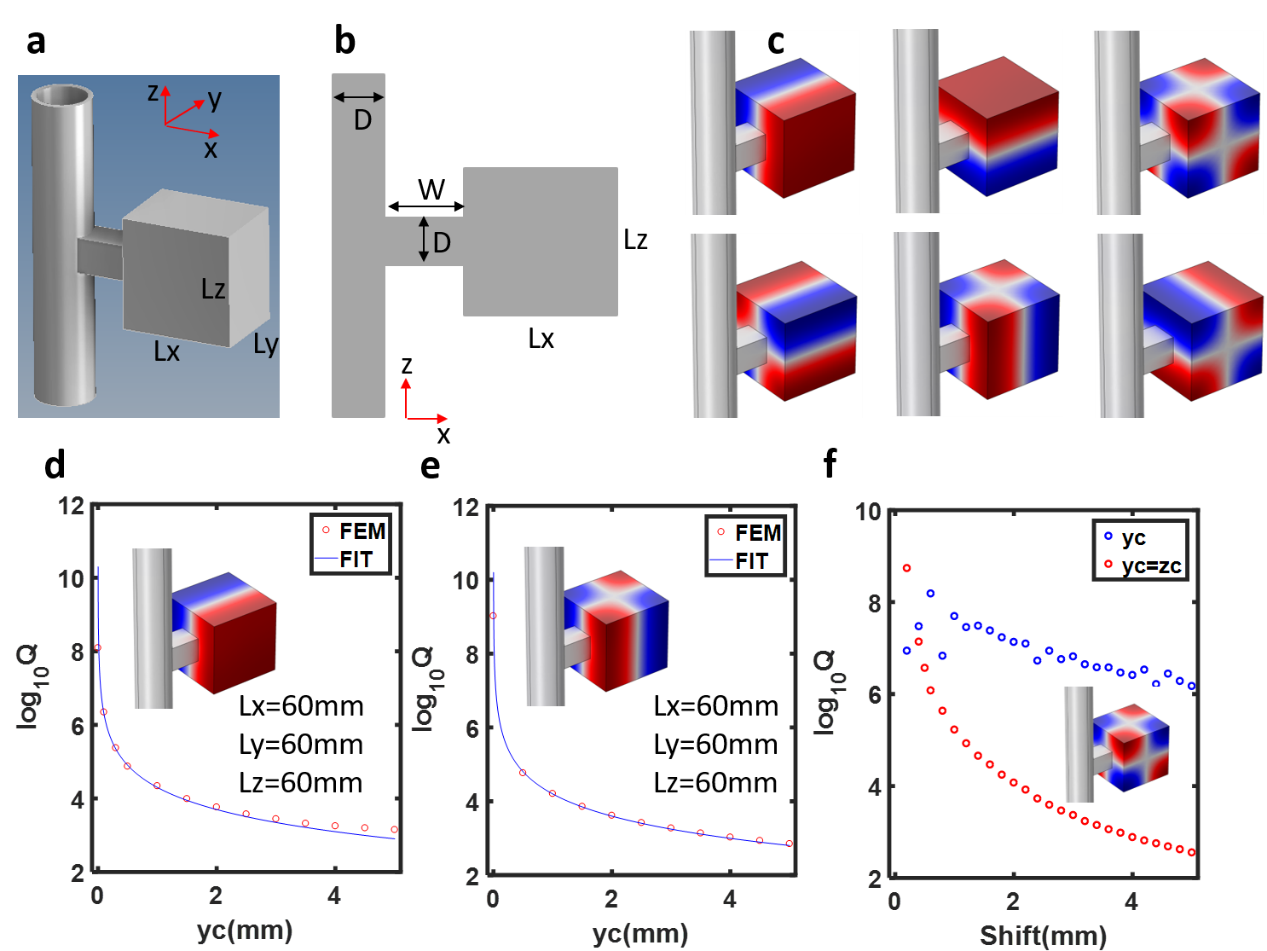


**Supplementary Figure 9. Symmetric protected BICs in cuboid resonator.** a-b, Schematic drawing of an open cuboid resonator. c, pressure distribution of BICs, including M_121_, M_112_, M_222_, M_122_, M_221_, M_212_. d, Q-factor vs yc for M_121_. e, Q-factor vs yc for M_221_. f, Q-factor vs yc and yc=zc for M_222_.


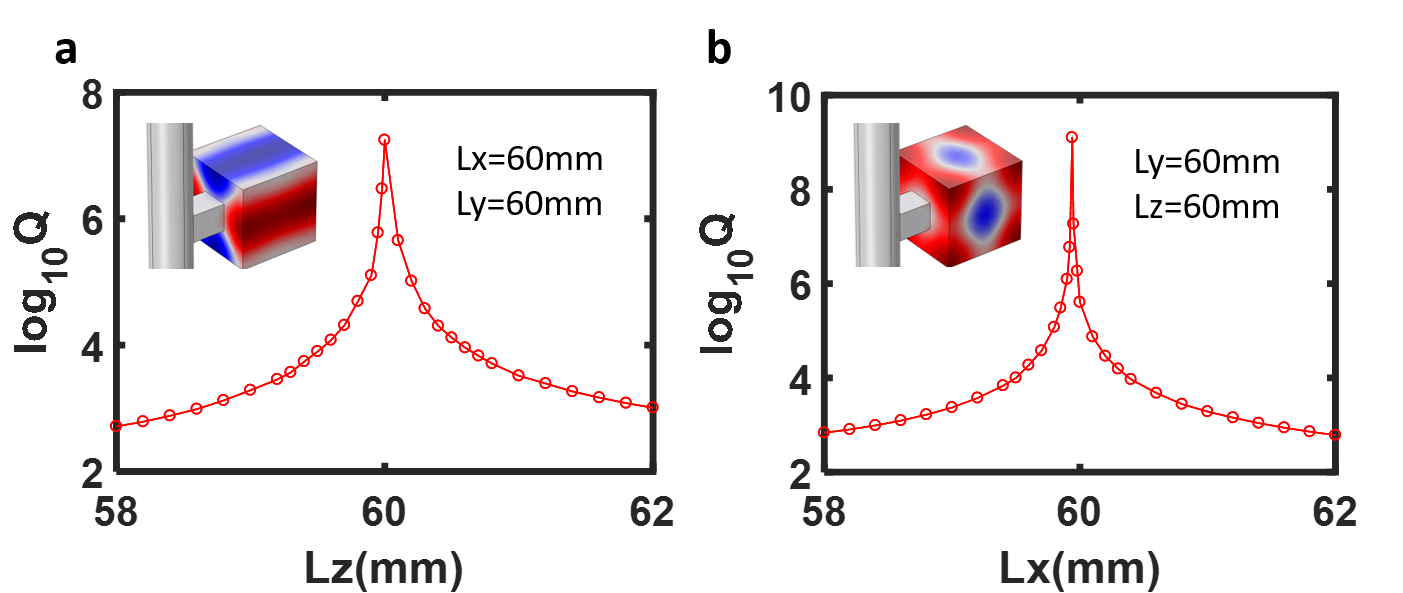


**Supplementary Figure 10. Mode interference induced BICs in cuboid resonator.** a, Q-factor vs Lz for M_131_. b, Q-factor vs Lx for M_113_.


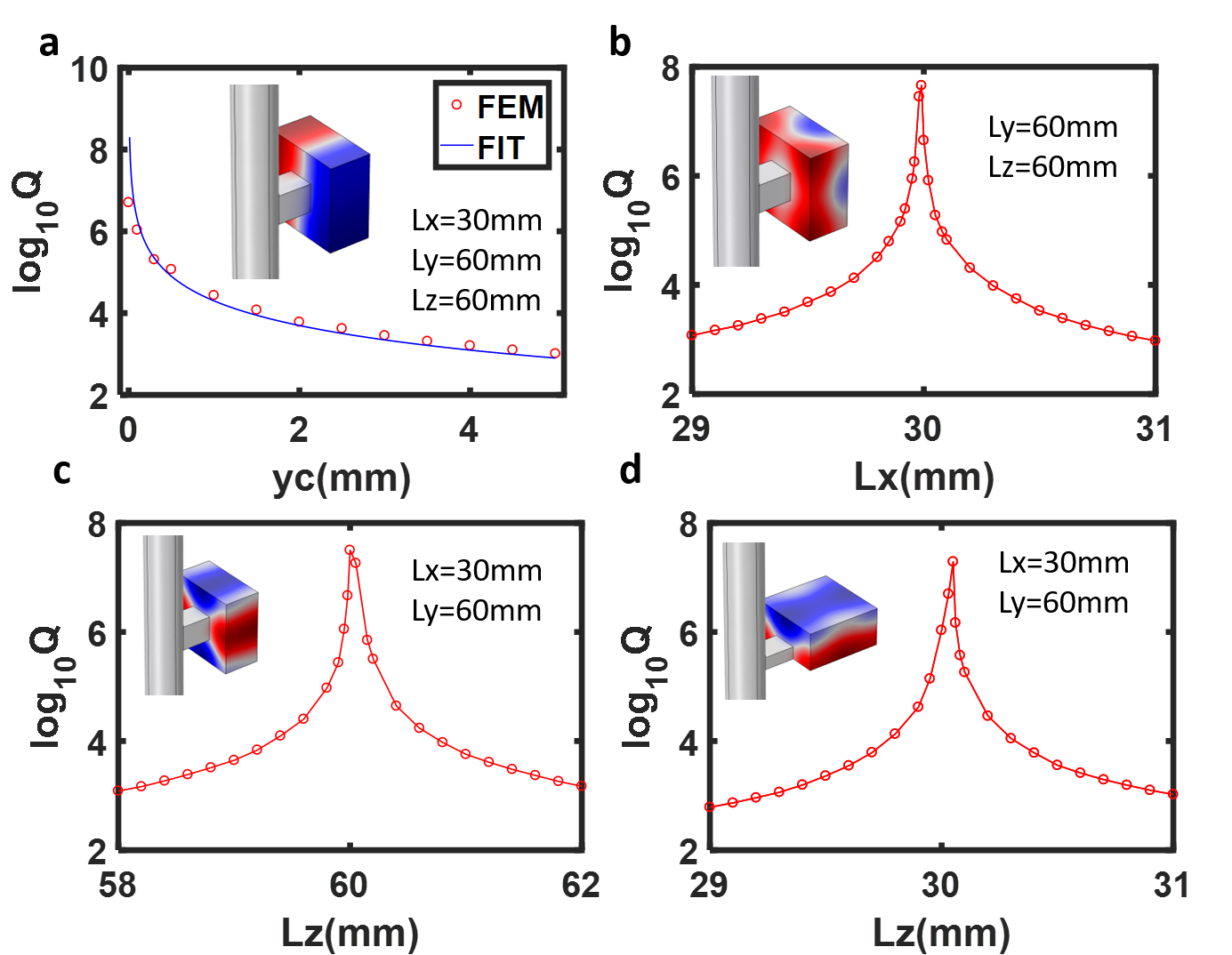


**Supplementary Figure 11. Mirror effect induced BIC in half cuboid resonator**. a, Q-factor of M_121_ vs yc. b, Q-factor of M_113_ vs Lx. c, Q-factor of M_131_ vs Lz. d, Q-factor of M_131_ vs Lz.


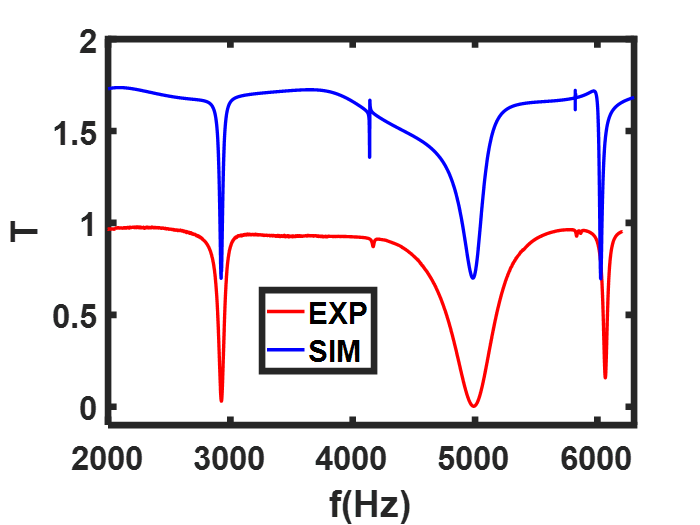


**Supplementary Figure 12.** Measured and simulated transmission spectrum for full resonator with yc=1mm.


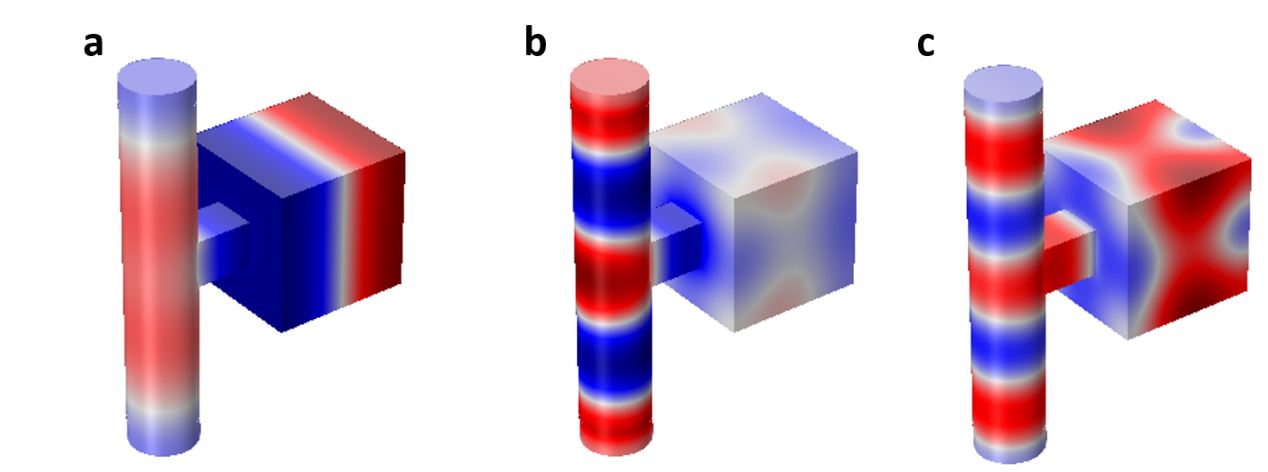


**Supplementary Figure 13.** Other leaky modes in transmission spectrum. a, pressure distribution of for mode with f=2926Hz. b, pressure distribution for mode with f=4990Hz. c, pressure distribution for mode with f=6063Hz


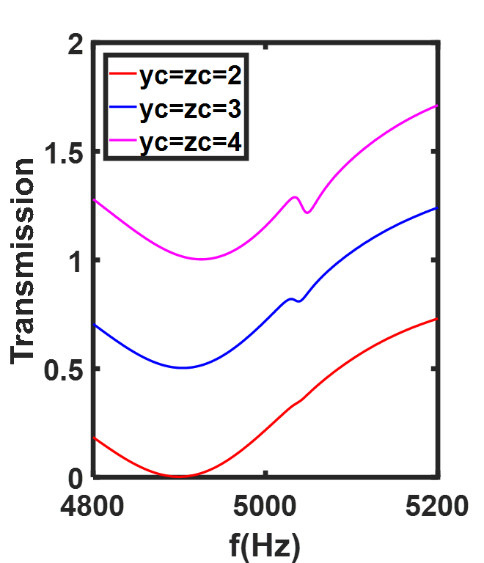


**Supplementary Figure 14.** Transmission spectrum for full resonator (Lx=Ly=Lz=60mm) with different yc=zc.


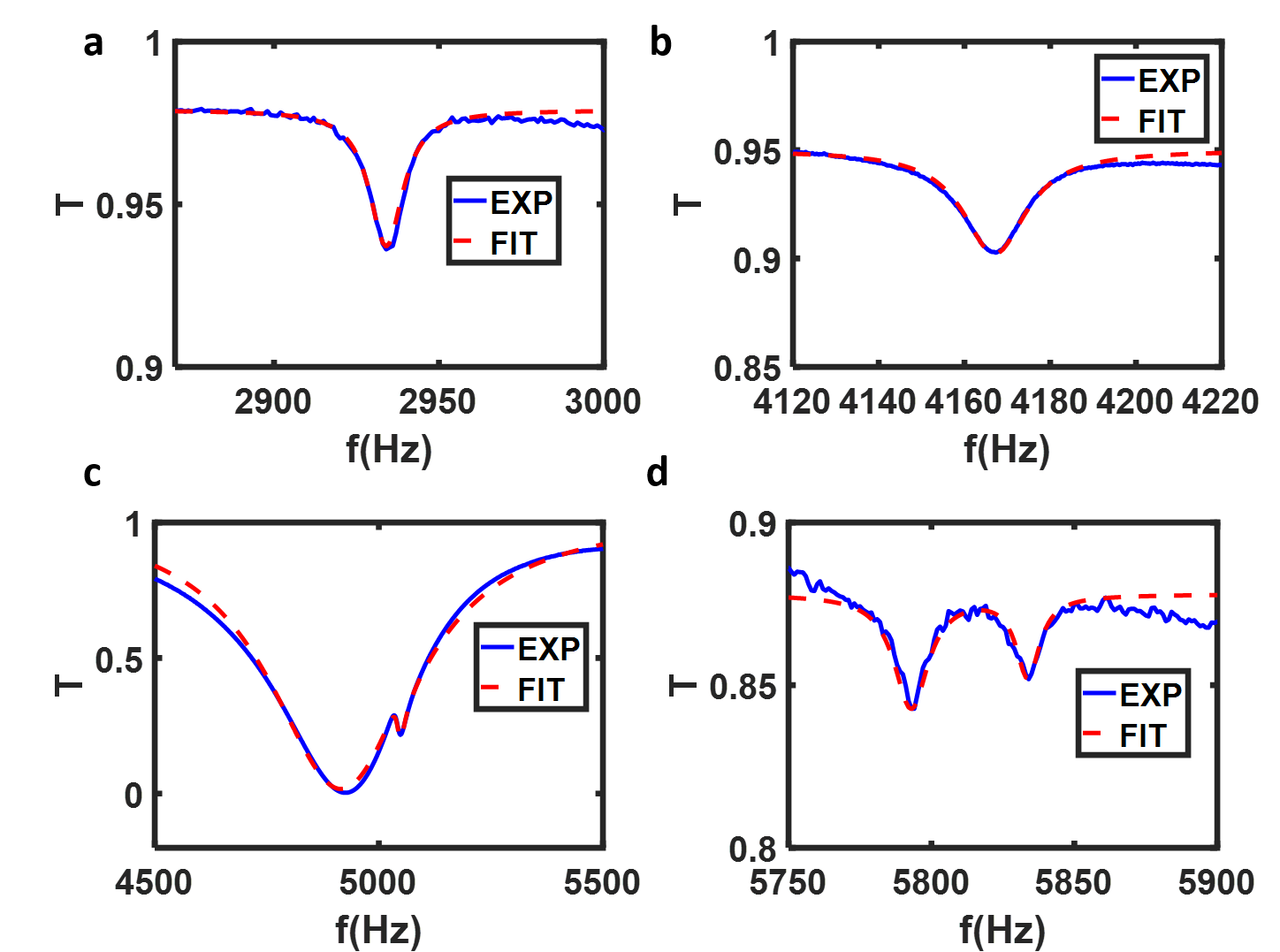


**Supplementary Figure 15. Fano fitting of transmission spectrum.** a, Fano fitting of transmission spectrum in half resonator with Lx=30mm, Ly=Lz=60mm, yc=1mm. b, Fano fitting of transmission spectrum in full resonator with Lx=Ly=Lz=60mm, yc=1mm. c, Fano fitting of transmission spectrum in full resonator with Lx=Ly=Lz=60mm, yc=zc=4mm. d, Fano fitting of transmission spectrum in full resonator with Lx=Ly=Lz=60mm, yc=zc=0mm.


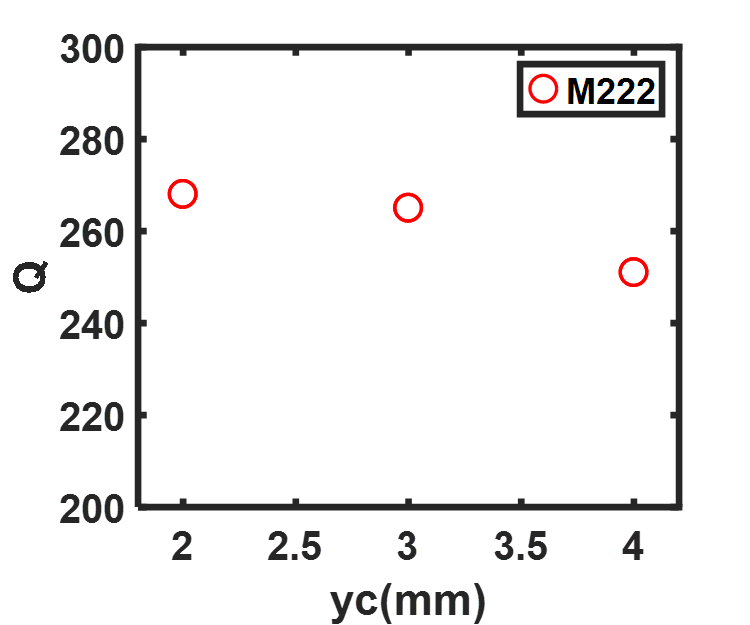


**Supplementary Figure 16.** Measured Q-factor of mode M222 as a function of center shift (yc=zc).


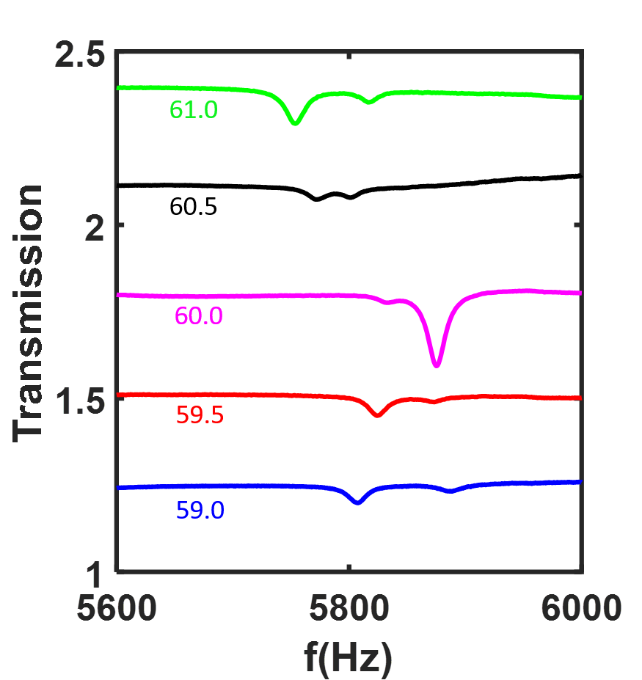


**Supplementary Figure 17.** Transmission spectra of half resonator (Lx=30mm and Ly=60mm) with different Lz.
